# Potassium permanganate elicits a shift of the external fish microbiome and increases host susceptibility to columnaris disease

**DOI:** 10.1186/s13567-015-0215-y

**Published:** 2015-07-15

**Authors:** Haitham H. Mohammed, Covadonga R. Arias

**Affiliations:** Aquatic Microbiology Laboratory, School of Fisheries, Aquaculture, and Aquatic Sciences, Center for Advanced Science, Innovation, and Commerce, 559 Devall Drive, Auburn, AL 36832 USA

## Abstract

The external microbiome of fish is thought to benefit the host by hindering the invasion of opportunistic pathogens and/or stimulating the immune system. Disruption of those microbial communities could increase susceptibility to diseases. Traditional aquaculture practices include the use of potent surface-acting disinfectants such as potassium permanganate (PP, KMnO_4_) to treat external infections. This study evaluated the effect of PP on the external microbiome of channel catfish and investigated if dysbiosis leads to an increase in disease susceptibility. Columnaris disease, caused by *Flavobacterium columnare,* was used as disease model. Four treatments were compared in the study: (I) negative control (not treated with PP nor challenged with *F. columnare*), (II) treated but not challenged, (III) not treated but challenged, and (IV) treated and challenged. Ribosomal intergenic spacer analysis (RISA) and pyrosequencing were used to analyze changes in the external microbiome during the experiment. Exposure to PP significantly disturbed the external microbiomes and increased catfish mortality following the experimental challenge. Analysis of similarities of RISA profiles showed statistically significant changes in the skin and gill microbiomes based on treatment and sampling time. Characterization of the microbiomes using 16S rRNA gene pyrosequencing confirmed the disruption of the skin microbiome by PP at different phylogenetic levels. Loss of diversity occurred during the study, even in the control group, but was more noticeable in fish subjected to PP than in those challenged with *F. columnare*. Fish treated with PP and challenged with the pathogen exhibited the least diverse microbiome at the end of the study.

## Introduction

Fish are in intimate contact with the aquatic environment which harbors pathogenic and opportunistic organisms [1]. As a result, cutaneous diseases are more common in fish than in terrestrial vertebrates [2] and the external epithelial surfaces are often the major route of entry for infectious agents in aquatic animals [3]. Skin and gills of fish are extremely important as the first line of defense against invasion by opportunistic pathogens and subsequent infections that may result in disease. In addition to being mechanical barriers, skin and gills represent a biologically active environment [4,5] that is colonized by a diverse, complex and dynamic microbial communities that constitutes the fish external microbiome [6-10]. A healthy microbiome exerts antagonistic effects against pathogens by competitive exclusion for nutrient and/or synthesis of antimicrobial compounds and promotes host homeostasis [11,12]. Suppression of pathogenic organisms by the resident microbiota has been reported in birds, fish, crustaceans, and other aquatic organisms [10,13,14]. Thus, preserving the integrity of the normal protective microbiome is key for excluding potential invaders and maintaining health [15].

Intensive production practices used in fish farms can result in environmental stressors such as low dissolved oxygen or high organic loads that favor opportunistic pathogens and are stressful to fish [16]. Moreover, the use of chemical treatments to control or prevent specific pathogens can alter the normal healthy fish microbiome making the fish more vulnerable to infections [17]. The effect of these intensive culture practices on the fish external microbiome is for the most part unknown. We hypothesized that the use of harsh chemicals as treatment against external bacterial, parasitic and fungal infections disrupts the skin and gill microbiome and increases susceptibility to opportunistic bacterial pathogens. To test our hypothesis, we chose to use PP (KMnO_4_), a potent oxidizing agent commonly used in aquaculture to treat external infections, and *Flavobacterium columnare* as the causative agent of columnaris disease, a very common bacterial infection in freshwater aquaculture farms.

Columnaris disease courses primarily as an external infection and the bacteria frequently attack the fins, skin, and gills of fish causing frayed fins, depigmented or ulcerated skin and necrotic gills [18,19]. Skin and gills are believed to be the point of entry and the primary site of infection for *F. columnare* [3,20] and bacterial competition is considered one of the factors determining the degree of the infection [21]. Previous studies have shown that survival and infectivity of *F. columnare* decline in presence of competitive bacteria species such as *Aeromonas hydrophila* (an opportunistic fish pathogen) and *Citrobacter freundii* (nonpathogenic to fish) [22] or when the density of *F. columnare* was too low relative to total bacterial counts [23]. Thus, it has been suggested that when *F. columnare* is present in low numbers, it may not be able to compete with other naturally occurring bacteria on the fish skin and gills [24].

To prove if PP altered the composition of the fish external microbiome and, subsequently, increased susceptibility to columnaris disease we applied culture-independent methods to characterize and compare the channel catfish (*Ictalurus puntactus*) external microbiome before and after exposure to PP and challenge with *F. columnare.* Our model has direct implications for commercial aquaculture as channel catfish is the main aquaculture species in the U.S. and is highly susceptible to columnaris disease. In addition, PP is routinely used in freshwater fish farms around the world to control external infections.

## Materials and methods

### Fish husbandry

Channel catfish fingerlings (*n* = 199, average weight ± SD was 15 ± 1.7 g and average length ± SD was 14.3 ± 0.7 cm) were purchased from Osage Catfisheries Inc. (Osage Beach, MO, USA) (the fish were inspected by University of Arkansas at Pine Bluff Fish Diseases Laboratory and found to be free of pathogens, Case ID#:PB11-233) and express shipped to the E. W. Shell Fisheries Center (EWSFC) at North Auburn Fisheries Experiment Station, Auburn, AL, USA. Fish were kept in a 250 gallons plastic tank supplied with dechlorinated city water for 4 weeks prior to the experiment. Fish were then transferred in aerated containers to the Aquatic Microbiology Laboratory (AML) located on main campus at Auburn University. Upon arrival to AML and prior to stocking in the glass aquaria/tanks, mucus, skin and gill samples of ten randomly caught fingerlings were sampled, examined following standard procedures [25] and proved culture negative for *F. columnare*. Before fish were transferred to the glass aquaria, DNA was extracted from the skin and gills of 9 randomly caught fingerlings from the stock tank (t_0_). Fish were then stocked into 12 tanks, 37 L each at a stocking rate of 15 fish/tank and maintained as previously described [26]. Water quality was monitored daily and parameters were maintained at 80 ppm alkalinity, 40 ppm hardness, 0.1 ppt salinity, 26 ± 1 °C, pH 7.7 ± 0.2 [mean ± SD], ammonia and nitrites were kept at non-detectable levels and a dark and light period of 12:12 h was maintained throughout the experiment. Fish were acclimated for 7 days before treatment with PP. Fish were fed daily to apparent satiation with commercial pellets, AQUAMAX Grower 400 (Purina Mills, Inc., St. Louis, MO, USA). All animal protocols were approved by the Auburn University Institutional Animal Care and Use Committee (IACUC number 2012–2141).

### Experimental design

**Figure 1:**
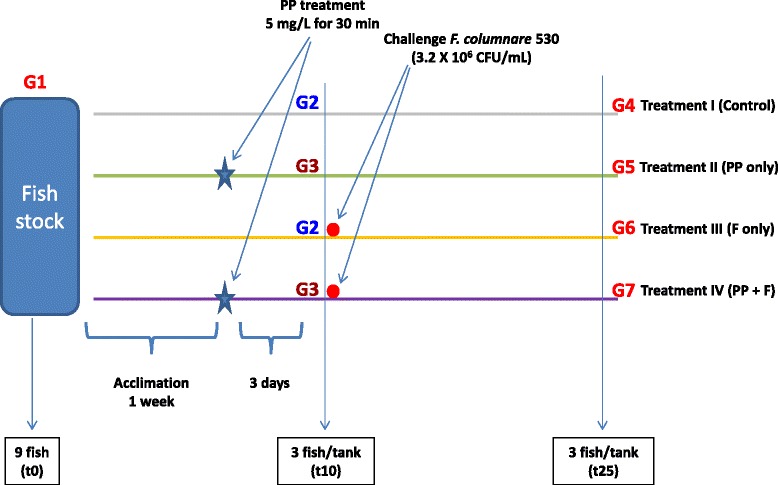
**Experimental design showing the different treatments, time points and groups used in the study.** (I) control = not treated nor challenged, (II) PP = treated with PP and not challenged, (III) F = not treated with PP and challenged with *F. columnare*, and (IV) PP + F = treated with PP and challenged with *F. columnare*. Treatments and DNA collection points (t_0_, t_10_, and t_25_) are indicated on the timeline. Groups (G1 to G7) are indicated on each treatment.

The study design is shown in Figure 1. Four treatments with 3 replicates each (replicate = tank) were set up as follows: (I) Non-treated non-challenged fish acted as controls (not exposed to PP and not challenged with *F. columnare*), (II) treated with PP and not challenged with *F. columnare*, (III) not treated with PP and challenged with *F. columnare*, and (IV) treated with PP and challenged with *F. columnare*. Tanks were randomized and assigned blindly to each treatment. For PP treatment, a dose of 5 mg/L above 15 min PP demand (PPD) of the tank water was applied [27,28]. PPD is a measure of the amount of PP required to react with organic matter in a 15 min time frame [29]. PPD was determined [29] prior to the treatment and the average was 0.4 mg/L. The final PP dose was calculated as the PPD (0.4 mg/L) + 5 mg/L. Two of the treatment groups (II and IV) were treated with PP for 30 min in buckets containing 5 L aerated water by adding 27 ml of the stock solution to each bucket (A stock PP solution was prepared by dissolving 1 g of PP in 1 L of water). Fish in treatments I and III were similarly handled but were not exposed to PP (received a sham treatment). At the conclusion of the 30 min treatment, fish were removed from the buckets and returned to their respective tanks. Fish were not fed during PP exposure, but were offered food afterwards. Fish were allowed 3 days of recovery time after exposure to PP and before challenge with *F. columnare*. Challenge with *F. columnare* was carried out as previously described [26]. Briefly, fish were exposed for 30 min to pathogenic strain ALG-00-530 (genomovar II) at a concentration in the challenge bath of 3.2 × 10^6^ CFU/mL. Fish in treatments I and II were similarly handled but sham challenged using sterile modified Shieh (MS) broth as inoculum in the challenge suspension. After the challenge, fish were removed from the challenge buckets, returned to their respective tanks and maintained under normal husbandry conditions. Fish were not fed on the challenge day, but were offered food on the next day after challenge and throughout the rest of the study. Fish were observed for clinical signs of columnaris disease and mortality was recorded twice daily. Columnaris infection was confirmed in moribund and dead fish by isolation of *F. columnare* as previously described [30].

### Sampling

Skin and gills were sampled for DNA extraction at time 0 (t_0_ = fish from stock tank), at time 10 days (t_10_ = three days after treatment with PP and immediately before the challenge) and at time 25 days (t_25_ = from the survivors at the end of the experiment). Three fish were sampled at each time point per tank except from the stock tank at t_0_ (9 fish were sampled) and from treatment IV (at the end of the experiment t_25_, all the fish died in a tank and in another tank, only 2 catfish survived). To analyze the data, we further subdivided the samples from the four treatments into seven groups based on designated time points (Figure 1). Group 1 (G1), samples from the stock tank at t_0_; Group 2 (G2), samples from treatments I&III (non-treated with PP) at t_10_; Group 3 (G3), samples from treatments II&IV (treated with PP) at t_10_; Group 4 (G4), samples from treatment I (system control) at t_25_; Group 5 (G5), samples from treatment II (treated with PP) at t_25_; Group 6 (G6), samples from treatment III (challenged with *F. columnare*) at t_25_; Group 7 (G7), samples from treatment IV (treated with PP and challenged with *F. columnare*) at t_25_.

### DNA extraction

All skin (*n* = 77) and gill (*n* = 77) samples for DNA extraction (<30 mg from each tissue) were taken from the tip of the lower lobe of the caudal fin and from the second right gill arch, respectively. To account for variability associated with DNA extraction and downstream nucleic acid analysis, three fish were sampled per tank at each sampling time. All samples were immediately subjected to DNA extraction using the DNeasy Blood & Tissue kit (Qiagen, Valencia, CA, USA) following manufacturer’s instructions (Total DNA from Animal Tissues, Spin-Column Protocol), including pretreatment with lysozyme for lysis of Gram positive bacteria. DNA was eluted with 100 μL elution buffer and was quantified using a NanoDrop *ND-1000* spectrophotometer (Thermo Scientific, Nanodrop Technologies, Wilmington, DE, USA).

### Ribosomal intergenic spacer analysis (RISA)

Extracted DNA was used as a template for RISA which was performed as previously described by Arias et al. (2006) with some modifications. The primer sequences ITS-FEub (5′-GTCGTAACAAGGTAGCCGTA-3′) and ITS-REub (5′-GCCAAGGCATCCACC-3′) were used for PCR amplification of the internal transcribed spacer region [31]. The PCR master mix contained 1x Taq buffer, 0.4 mM dNTPs (Promega, Madison, WI, USA), 2 mM MgCl_2_, 0.4 μM ITS-FEub primer, 0.2 μM ITS-REub primer, 2 μM ITS-REub labeled primer, 1 U of Taq polymerase (5 PRIME, Inc., Gaithersburg, MD, USA), and 10 ng of template DNA in a final volume of 50 μL. The samples were amplified in a PTC-200 DNA-Engine thermocycler (PTC-200, MJ Research, Watertown, MA, USA) and the PCR conditions were as follows: initial denaturation at 94 °C for 3 min, followed by 30 cycles of 94 °C for 45 s, 55 °C for 1 min, and 68 °C for 2 min, with a final extension step at 68 °C for 7 min. To prepare samples for gel loading, 10 μL of each PCR product were diluted with 10 μL AFLP® Blue Stop Solution (LI-COR). Diluted samples were denatured at 95 °C for 5 min followed by quick cooling (to prevent reannealing) prior to gel loading (0.6 μL of sample was loaded into each well). PCR products were electrophoresed on a LI-COR 4300 DNA Analyzer (LI-COR Biosciences, Lincoln, NE, USA) following manufacturer’s instructions. RISA gel images in TIFF format were exported to Bionumerics v. 7 (Applied Maths, Austin, TX, USA) and were analyzed as previously described [32].

### Pyrosequencing

To identify the predominant bacterial species on catfish skin, DNA of 21 skin samples (3 samples per group) were randomly selected for sequencing. The variable V1-V3 region of the 16S rRNA gene was amplified by PCR using the universal Eubacterial primer set 27 F (5′-AGRGTTTGATCMTGGCTCAG-3′) and 519R (5′-GWATTACCGCGGCKGCTG-3′) as described before [33]. Amplicons were then subjected to Roche 454 FLX titanium sequencing following manufacturer’s guidelines. The resulting sequences were processed using a proprietary analysis pipeline (MR DNA, Shallowater, TX, USA). Barcodes and primers were removed from the sequences, followed by removal of short sequences <200 base pairs in length, ambiguous base calls, and homopolymer runs longer than 6 base pairs. Afterwards, sequences were denoised and chimeras and singleton sequences were removed. Operational taxonomic units (OTUs) were defined at a cutoff value of 3% divergence (97% similarity) in agreement with the current accepted prokaryote species concept [34-39]. Final OTUs were taxonomically assigned using BLASTn against the Greengenes database [40]. Since species richness and evenness can be compared only between samples with equal sample sizes [41], we randomly normalized the sequences so as to standardize to the samples with the least number of sequences obtained (*N* = 1813) (the number of reads for each sample was normalized by randomly subsampling from the larger sample to the number of reads of the smallest one). Rarefaction curves, Good’s coverage, abundance-based coverage estimation (ACE), Chao1, Shannon evenness, and shared OTUs based on defined OTUs were generated using Mothur v.1.33.3 package [42]. Sample-by-OTU abundance data matrices from mothur were subsequently transposed and multivariate analysis was performed with the PRIMER 6 (Plymouth Routines In Multivariate Ecological Research) software package.

### Data analyses

Bionumerics v. 7 (Applied Maths) was used to process RISA images. Following normalization and background subtraction with mathematical algorithms, similarity levels between fingerprints were calculated by Pearson product–moment correlation coefficient. Cluster analysis was performed according to Arias et al. using the Unweighted Pair Group Method with Arithmetic Mean (UPGMA) [32]. Multidimensional scaling (MDS) was performed using optimized positions to visualize the similarities or dissimilarities of the samples. Analysis of similarities (ANOSIM) was run on the similarity matrix generated from Bionumerics using PRIMER v6 (Primer-E Ltd, Plymouth, UK). Mortality data was analyzed using analysis of variance (ANOVA) with general linear model (PROC GLM) followed by Tukey’s Studentized Range (HSD) test for all-pairwise comparisons to determine significant (*p* < 0.05) differences between the mean mortality of the different treatments (SAS Institute, Cary, NC.). A one-way ANOVA was performed on all diversity indexes, followed by a Tukey’s post hoc test where significance (*P* < 0.05). A genera abundance table was loaded into PRIMER v6 [43] and similarity percentages (SIMPER) analysis was performed to determine the genera responsible for differences between groups. Cut-off for low contributions was set at the default 90%.

## Results

### Mortality

**Figure 2:**
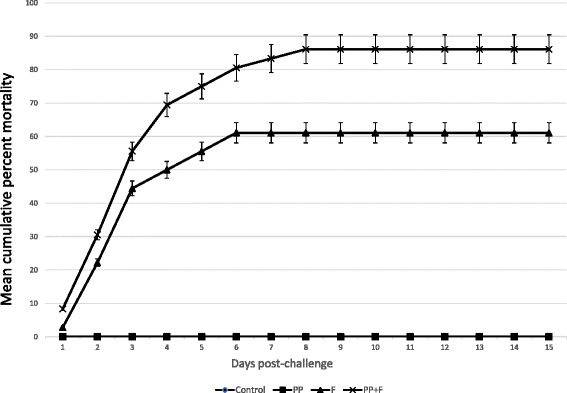
**Mean cumulative percent mortality of channel catfish challenged with** ***Flavobacterium columnare.*** (I) control = not treated nor challenged, (II) PP = treated with PP and not challenged, (III) F = not treated with PP and challenged with *F. columnare*, and (IV) PP + F = treated with PP and challenged with *F. columnare*. (Note: treatments I and II had 0% mortality, so the mortality curves are superimposed).

The mean cumulative percent mortality of the four treatments is shown in (Figure 2). Control (treatment I) and PP treated but non-challenged fish (treatment II) did not show any mortality throughout the experiment. Fish non-treated with PP and challenged with *F. columnare* (treatment III) had a mean percent mortality of 61.1 ± 1.5 (SD), which was significantly different (*P* < 0.05) from the mortality observed in fish treated with PP and challenged with *F. columnare* (treatment IV) that was 86.1 ± 1.5 (SD). Mortalities of both challenged treatments significantly differed from that of the non-challenged treatments (0%). Channel catfish fingerlings in challenged tanks (treatments III and IV) exhibited clinical signs typical of columnaris disease. *F. columnare* was isolated from skin lesions, gills and kidneys of dead or moribund fish. Anecdotal observations at day 1 post-challenge, suggested that fish treated with PP and challenged with *F. columnare* (treatment IV) were more lethargic with rapid opercular movement than those challenged but not PP treated (treatment III). Mortality persisted for 8 days with the majority of fish deaths occurring on days 2 and 3 post-challenge. The study was concluded on day 15 after 7 consecutive days without mortalities.

### RISA

**Figure 3:**
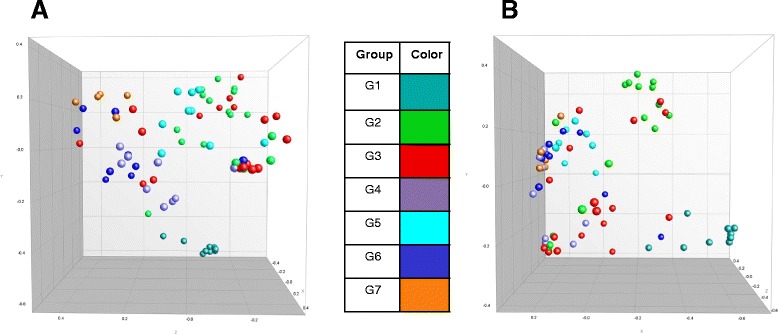
**Multidimensional scaling (MDS) plot of skin (panel** **A** **) and gill (panel** **B** **) samples.** The similarity matrix obtained was used to compare RISA fingerprints based on groups. Distance between entries represents graphical dissimilarities obtained from the similarity matrix.

A total of 154 (77 skin & 77 gill) samples were analyzed by RISA representing all seven groups (see Figure 3). RISA profiles averaged 25 bands that ranged in size between 50 to 700 bp. Similarities between microbial community profiles ranged from a maximum of 99% to a minimum of 17.5% based on Pearson correlation coefficient analysis followed by UPGMA clustering. For better visualization of the clusters observed by RISA, MDS was used to display skin and gills microbiome profiles using the variables treatment, time, tissue and group. Figure 3 shows the MDS plot of skin and gill samples based on group ascription. ANOSIM directly compared the clusters based on the following variables: treatment (I through IV), time (t_0_, t_10_, t_25_), tissue (skill and gill) and group (G1 through G7). Samples clustered significantly (*p* = 0.001) by all factors considered, although there was some overlap among them (Table 1). The least significant factor for the cluster separation was tissue (skin or gill) with an R value of 0.093. Separation was most significant when samples were assigned to clusters based on group with an R value of 0.387 and 14 out of 21 pairwise comparisons were significant while only 7 were not significant. The R values for treatment and time were 0.214 and 0.304, respectively. Seven out of 10 and 10 out of 10 pairwise comparisons were significantly different by treatment and by time, respectively. These global R values indicate that RISA-based clusters are significantly correlated with all the factors although group (group = treatment + time combined) was the most significant variable and played the main role determining the change in composition of the skin and gill microbiome.

**Table 1:** Analysis of similarities (ANOSIM) values obtained when RISA profiles were ascribed to the variables tested in the study

Variable	Global R	*P* value	# Significant pairwise comparisons
Tissue	0.093	0.001	-
Treatment	0.214	0.001	7 out of 10
Time	0.304	0.001	3 out of 3
Group	0.387	0.001	14 out of 21

### Pyrosequencing

**Figure 4:**
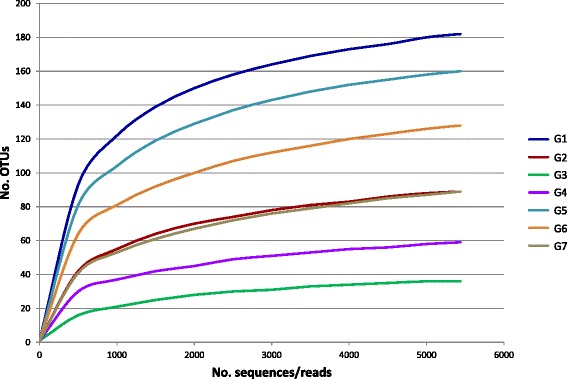
**Rarefaction curves of skin samples when OTUs where defined at 97% sequence similarity.** Samples were standardized to the least number of sequences obtained.

Twenty one skin samples, 3 replicates per group, were subjected to 16S rRNA gene pyrosequencing. No gill samples were sequenced as diversity on fish gills was previously found to be lower than that on fish skin [44-46] and our skin and gill RISA results were in agreement. Pyrosequencing yielded a total of 236 697 bacterial sequences and 483 OTUs. After sample normalization, 38 073 sequences and 454 OTUs were included in the analysis. Sequence coverage was ≥98% in all sequenced samples (Good’s coverage, Table 2). Rarefaction curves (Figure 4) confirmed that G3 (3 days post-treatment with PP) was the group with the least diverse bacterial population. G1 (fish prior tank stocking) displayed the most diverse microbiome. Total expected richness as calculated by ACE and Chao1 was significantly different between groups and the Shannon evenness index was significantly different as well (Table 2).

**Table 2:** Diversity indices as calculated by MOTHUR software (ver. 1.33.3). Operational taxonomic units (OTUs) were defined at 97% sequence similarity. Significance among total values for each fish species was determined by a one-way anova followed by Tukey’s post hoc test. Within a column, different superscript letters means significant difference (anova: *p* < 0.01)

Group	Sobs^a^	Good’s coverage	ACE^b^	Chao1	Shannon evenness
G1	96 ^A^	0.988	110 ^AB^	113 ^AB^	0.731 ^AB^
G2	38 ^C^	0.996	44 ^C^	44 ^C^	0.659 ^BC^
G3	23 ^C^	0.996	29 ^C^	27 ^C^	0.191 ^D^
G4	93 ^AB^	0.986	123 ^A^	125 ^A^	0.760 ^A^
G5	56 ^BC^	0.991	71 ^BC^	68 ^BC^	0.636 ^C^
G6	112 ^A^	0.985	132 ^A^	131 ^A^	0.768 ^A^
G7	42 ^C^	0.994	54 ^C^	52 ^C^	0.584 ^C^

^a^, Sobs, the total number of species observed in the community.

^b^, ACE, abundance-based coverage estimation.

**Figure 5:**
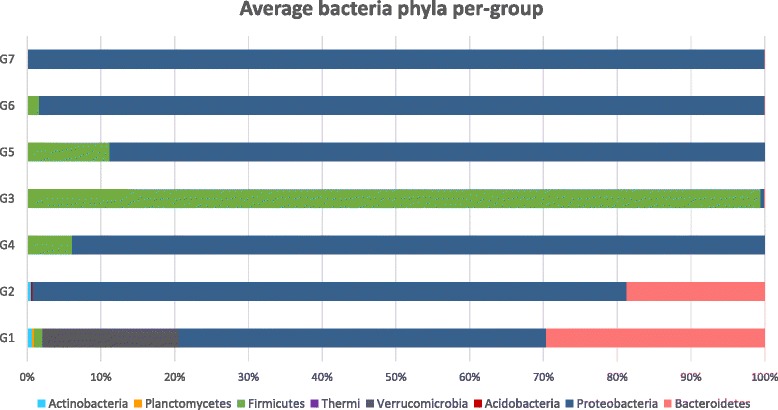
**Bacteria phyla composition for each group, representing average of all replicates, obtained by pyrosequencing.** Bacterial diversity at the phylum level based on pyrosequencing of 16S rRNA gene showing the differences in the skin microbiome structure between groups and the percent of detected sequences belonging to the different bacterial phyla in each group.

When sequences were ascribed at the phylum level, each group returned a unique bacterial composition. Eight bacterial phyla (Actinobacteria, Planctomycetes, Firmicutes, Thermi, Verrucomicrobia, Acidobacteria, Proteobacteria and Bacteroidetes) were identified from the skin samples of all groups (Figure 5). Proteobacteria accounted for 73.1% of all sequences obtained, whereas, Firmicutes represented 17.1% of the total sequences. Other less common phyla like Bacteroidetes, Verrucomicrobia and Actinobacteria formed 6.9%, 2.6% and 0.2%, respectively. The phylum Proteobacteria was the most predominant phylum in six groups and comprised the majority of all sequences (49.8% in G1, 80.5% in G2, 93.9% in G4, 88.8% in G5, 98.4% in G6 and 99.9% in G7) while in G3, the phylum Firmicutes was the most abundant phylum forming 99.5% of all sequences. Bacteroidetes was identified in varying levels in five groups (29.6% in G1 and 18.7% in G2, 0.05% in G3, 0.003% in G6 and 0.002% in G7). The less common phyla varied in abundances between groups. Planctomycetes, Thermi and Verrucomicrobia were unique to G1 (0.3%, 0.1% and 18.4%, respectively). Acidobacteria was identified merely in G2 (0.2%). Sequences for Actinobacteria were detected only in G1 and G2 representing 0.7% and 0.5%, respectively.

The skin microbiome of all groups was composed of a total of 105 genera; only genera accounting for more than 5% of all identified sequences in at least one group are presented in Table 3. Proteobacteria was represented by many genera but the most common were *Aeromonas, Vogesella, Stenotrophomonas, Klebsiella, Trabulsiella*, *Citrobacter*, *Enterobacter*, *Rheinheimera, Pseudomonas, Acinetobacter* and *Herbaspirillum.* The majority of all Firmicutes sequences correspond to members of the genus *Bacillus*. Bacteroidetes was represented by the genera *Chryseobacterium* and *Runella.* The genus *Flavobacterium* accounted for only 0.4% of all genera identified in all groups. Verrucomicrobia and Actinobacteria were mostly represented by *Puniceicoccaceae* and *Propionibacterium*, respectively. Out of the 105 genera identified, only 3 genera (*Enterobacter, Raoultella and Citrobacter*) were shared between the microbiome of the seven groups, suggesting significant dissimilarity in the bacterial composition of the skin between groups at the genus level. Predominant genera varied between groups with *Bacillus* being the most abundant genus in G3, *Aeromonas* in G4 and G6, *Vogesella* in G5, *Stenotrophomonas* in G2 and *Klebsiella* in G7. Other relatively abundant genera included *Trabulsiella*, *Citrobacter* and *Enterobacter* in G7, *Chryseobacterium* in G1 and G2, *Rheinheimera* in G2, and *Puniceicoccaceae*, *Pseudomonas*, *Acinetobacter*, *Runella* and *Herbaspirillum* in G1.

**Table 3:** Genus identity of sequences represented by percentage from the total sequences. Only genera accounting for more than 5% of sequences in at least one group are displayed

Genus	G1	G2	G3	G4	G5	G6	G7
*Bacillus*	0	0	99.469	6.104	11.236	1.613	0
*Aeromonas*	0.593	0.004	0.008	91.264	0	80.043	0
*Vogesella*	0.477	0.132	0	0	76.524	1.935	0.004
*Stenotrophomonas*	0.542	65.116	0.320	0	0.024	0	0.104
*Klebsiella*	0	0.189	0.004	0.036	5.041	5.177	41.45
*Trabulsiella*	0	0.057	0	0	3.258	2.599	26.952
*Chryseobacterium*	13.163	18.145	0.0328	0	0	0	0.002
*Puniceicoccaceae*	17.936	0	0	0	0	0	0
*Citrobacter*	0.051	0.552	0.007	0.260	1.724	2.685	15.273
*Enterobacter*	0.324	0.060	0.002	0.032	1.679	1.909	12.293
*Rheinheimera*	0.358	8.904	0	0	0	0	0
*Pseudomonas*	8.555	1.984	0.066	0	0.002	0	0
*Acinetobacter*	8.546	0.399	0.004	0	0	0	0
*Runella*	8.534	0	0	0	0	0	0
*Herbaspirillum*	5.191	0.208	0	0	0	0	0

**Figure 6:**
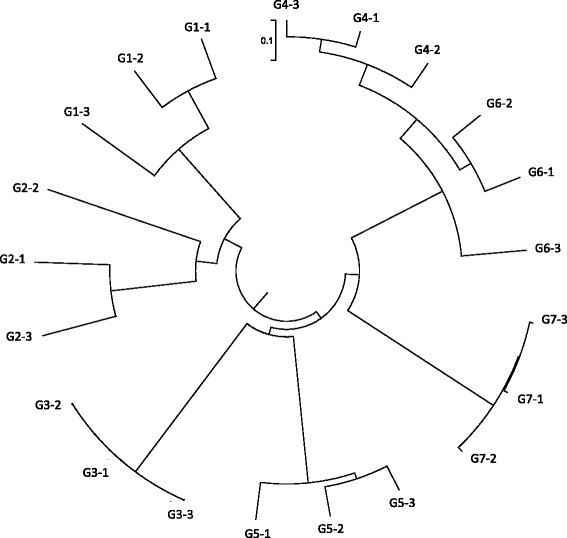
**A dendrogram illustrating the hierarchical arrangement of the sequenced samples showing all replicates per group.** The scale bar on the dendrogram represents the percentage of dissimilarity between two samples.

Similarity Percentage (SIMPER) analysis by bacterial genera between replicates (within each group) showed high similarities within group. Figure 6 summarizes the clustering analysis of all 21 skin samples analyzed. Conversely, SIMPER analysis showed high pairwise dissimilarities between groups (Table 4). The majority of the differences between groups were due to different relative abundances of the genera *Stenotrophomonas*, *Chryseobacterium*, *Puniceicoccaceae*, *Bacillus*, *Aeromonas*, *Klebsiella*, *Trabulsiella* and *Vogesella* (Table 4). Based on genus composition, SIMPER analysis indicated that G3 and G7 were the most dissimilar (99.94%), followed by G2 and G4 (99.86%), while G6 and G4 were the least dissimilar (26.62%).

**Table 4:** SIMPER analysis between groups showing pairwise dissimilarities and main genera contributing to dissimilarity

Average dissimilarity between groups	Bacteria genus	Group I average abundance	Group II average abundance	% Contribution to dissimilarity
G1 & G2 = 90.98	*Stenotrophomonas*	15	2132	11.63
	*Chryseobacterium*	1147	1519	10.27
	*Puniceicoccaceae*	1684	0	9.25
G1 & G3 = 99.75	*Bacillus*	0	9072	45.47
	*Puniceicoccaceae*	1684	0	8.44
G2 & G3 = 99.61	*Bacillus*	0	90.72	45.54
	*Stenotrophomonas*	2132	10	10.66
G1 & G6 = 99.18	*Puniceicoccaceae*	1684	0	8.49
	*Chryseobacterium*	1147	0	5.78
	*Aeromonas*	0	971	4.89
G2 & G6 = 99.35	*Stenotrophomonas*	2132	0	10.73
	*Chryseobacterium*	1519	0	7.64
G3 & G6 = 98.39	*Bacillus*	9072	142	45.38
	*Aeromonas*	0	971	4.93
G1 & G7 = 99.81	*Klebsiella*	0	3811	19.09
	*Puniceicoccaceae*	1684	0	8.44
	*Trabulsiella*	0	1387	6.95
G2 & G7 = 99.12	*Klebsiella*	13	3811	19.16
	*Stenotrophomonas*	2132	4	10.74
G3 & G7 = 99.94	*Bacillus*	9072	0	45.39
	*Klebsiella*	0	3811	19.06
G6 & G7 = 87.65	*Klebsiella*	474	3811	19.04
	*Trabulsiella*	144	1387	7.09
G1 & G5 = 99.44	*Vogesella*	10	2071	10.36
	*Puniceicoccaceae*	1684	0	8.47
G2 & G5 = 99.38	*Stenotrophomonas*	2132	0	10.73
	*Vogesella*	2	2071	10.41
G3 & G5 = 88.74	*Bacillus*	9072	1024	45.34
	*Vogesella*	0	2071	11.67
G6 & G5 = 90.21	*Vogesella*	50	2071	11.20
	*Aeromonas*	971	0	5.38
G7 & G5 = 87.97	*Klebsiella*	3811	453	19.09
	*Vogesella*	0	2071	11.77
G1 & G4 = 99.40	*Puniceicoccaceae*	1684	0	8.47
	*Chryseobacterium*	1147	0	5.77
G2 & G4 = 99.86	*Stenotrophomonas*	2132	0	10.68
	*Chryseobacterium*	1519	0	7.61
G3 & G4 = 93.90	*Bacillus*	9072	559	45.33
	*Aeromonas*	0	1079	5.75
G6 & G4 = 26.62	*Klebsiella*	474	2	8.86
	*Bacillus*	142	559	8.57
G7 & G4 = 99.68	*Klebsiella*	3811	2	19.11
	*Trabulsiella*	1387	0	6.95
G5 & G4 = 95.29	*Vogesella*	2071	0	10.87
	*Aeromonas*	0	1079	5.66

## Discussion

In the aquatic environment, both saprophytic and pathogenic organisms can infect fish when the conditions suit favorable for their multiplication [47]. However, under normal conditions, fish use a repertoire of innate and specific defense mechanisms to maintain healthy status and defend themselves against potential invaders [48]. The microbiome is now considered an essential extra organ of the host, and recent studies using gnotobiotic animals have shown the profound impact of bacteria on the anatomical, physiological and immunological development of the host [49,50]. Therefore, colonization of the fish surface by a healthy microbiome results in a protective barrier that enhances host fitness [15,51-54]. The microbiome can protect the host by outcompeting pathogens for living space, adhesion sites, energy and essential nutrients, or by producing inhibitory compounds and enhancing the immune response [55,56]. Disturbance of these functions by dysbiosis (an imbalanced or disrupted microbiota) may contribute to development of diseases. Stressful settings such as those occurring under intensive aquaculture production induce dysbiosis to the healthy fish microbiome, thus allowing pathogens to establish infections [17].

Our results show that PP treatment dramatically altered the community composition of the catfish external microbiome, as G3 (3 days post-exposure to PP) had the least diverse microbiome in terms of species richness. Furthermore, the phylum Proteobacteria was the predominant phylum on the skin microbiome of all groups except G3, which was dominated by the phylum Firmicutes (99.5% of all OTUs). This disruption in microbiome structure was correlated with a significant increase in mortality of fish treated with PP (86.1%) compared to those with intact external microbiome (61.1%) after pathogen exposure. Hence, dysbiosis of the external microbiome significantly increased catfish susceptibility to columnaris disease. This increase in susceptibility could be attributed to chemical injuries induced by exposure to PP; however, fish were allowed to recover from PP exposure for 3 days prior challenge. Previous studies reported that exposure to PP at therapeutic dose (as the one used in this study) can cause mild hypertrophy and spongiosis in gills but channel catfish recovered within 48-h post-treatment [57]. Similarly, when channel catfish were granted 3 days between physical injury and *F. columnare* exposure, regardless of the method of injury, no mortality was reported [58]. In our study, we could not separate the negative effect of PP on the external tissues from its effect on the external microbiome. However, based on previous studies [57], the integrity of the external tissues was restored soon after PP treatment while, based on our results, the microbiome was not. Therefore, the observed increase in susceptibility to bacterial infection is likely due to disruption of the normal beneficial microbiome caused by exposure to PP.

The phylum Proteobacteria dominated the skin microbiome of channel catfish, followed by the phylum Firmicutes, which was in agreement with previous studies on bacterial communities associated with fish skin in other species, regardless of the method used for identification [5,9,10,17,59]. After PP treatment, the external microbiome dramatically changed and all Proteobacteria were eliminated and substituted by Firmicutes. It was expected that Proteobacteria and other Gram-negative bacteria were less resistant to the action of PP than Gram-positive bacteria. A previous study showed that up to 32 mg/L PP is needed to reduce *Bacillus* sp. viable cells by 99% [60], a dose much higher than the one used in this study. Interestingly, members of the phylum Proteobacteria (*Aeromonas*, *Citrobacter*, *Pseudomonas* and *Luteimonas*) that were removed by PP treatment and replaced by Firmicutes, have shown antagonism to *F. columnare* in earlier studies [22,24,61,62]. On the other hand, although most probiotics proposed as biological control agents in aquaculture belong to the phylum Firmicutes, (*Bacillus*, *Lactobacillus*, etc.) [11], a thorough literature review revealed no antagonism between any Firmicutes (mainly *Bacillus*) and *F. columnare*. Our findings suggest that the observed shift from a “Proteobacteria dominated” to a “Firmicutes dominated” external microbiome results in the loss of key antagonistic species against *F. columnare*.

The variable “group” (Group = treatment + time combined) was the most influential factor affecting the skin microbiome composition. Each group presented a significantly distinct microbiome with a fairly low sample-to-sample variability within each group. At the phylum level, G1 displayed the most diverse microbiome with 7 out of 8 phyla found in the study present in this group. Interestingly, the microbiome composition differed significantly over the time during the study period even in the control treatment. Groups G2 and G4 significantly differed from G1 and from each other even though no treatment was applied to those fish except for handling. The phylum Verrucomicrobia was present in G1 but was not detected in G2. While the numbers of Bacteriodetes were significantly reduced from G1 to G2, the numbers of Proteobacteria increased. This trend continued over time and at day 25, control group G4 was overwhelmingly dominated by Proteobacteria (93.9%).

It is well known that moving fish is a source of stress and disease outbreaks are not uncommon after fish had been handled [63-65]. However, this is the first report in where significant changes in the external microbiomes of fish that were transferred between apparently similar environments have been documented. Our group has previously shown that skin microbiome is species-specific [9] but environmental factors and resident bacteria within an ecological niche can alter the bacterial communities associated with skin and mucus [17,44,66].

Differences in external microbiomes based on time were more apparently between G3 and G5 where the only difference between groups was sampling time after treatment with PP. For G3 at t_10_, Firmicutes represented 99.5% of the bacterial phyla percentages while Proteobacteria were 0.4%. At t_25_, G5 was dominated by Proteobacteria (88.2%) and the percentage of Firmicutes decreased drastically to 11.2%. Normally, the skin microbiome is dynamic and its composition fluctuates/shifts (community adaptation) over time and in response to changes in the environmental conditions [17,59,67-70]. Groups subjected to only one treatment (G5 = PP and G6 = pathogen) seemed to recover and shared a similar microbiome to that found in control group G4. Conversely, after two treatments (PP and pathogen) group G7 external microbiome was entirely reduced to Proteobacteria.

Overtime Proteobacteria became the predominant phylum regardless of the composition at earlier time points. However, not all microbiomes dominated by Proteobacteria were comprised of the same genera. At the genus level, only 3 genera were present in all the groups out of 105 total genera identified and genera abundance within Proteobacteria differed dramatically between groups (Table 3). The microbiome of fish in treatment IV (PP treatment) was dominated by the genera *Bacillus* before challenge at t_10_ (G3) and by *Klebsiella*, *Trabulsiella*, *Citrobacter* and *Enterobacter* at t_25_ (G7). The microbiome of fish in treatment III (*F. columnare* treatment) was dominated by the genera *Stenotrophomonas* before challenge at t_10_ (G2) and by *Aeromonas* at t_25_ (G6). This substantial difference in genera abundance between PP-treated fish compared to the untreated fish microbiome may have determined the increased susceptibility to *F. columnare* infection. However, further studies under field conditions are needed to fully understand the resilience of the fish microbiome to PP treatments in aquaculture ponds. Future studies should explore if manipulation of the fish microbiome by using pre- or probiotics will lead to a more natural and sustainable approach to prevent columnaris disease in aquaculture farms.

In conclusion, our data proved that harsh chemical treatments commonly used in fish farms induce dysbiosis to the fish’s healthy microbiome, reducing the numbers of beneficial bacteria and potentially increase susceptibility to pathogens. Our study emphasizes the fundamental importance of maintaining the integrity of the external microbiome as front-line defender against opportunistic pathogens like *F. columnare*. In the context of mutualism, fish in aquaculture could benefit from manipulating the composition of their external microbiome in order to decrease the incidence of columnaris disease. To the best of our knowledge, this is the first study to identify the skin microbiome composition of channel catfish. Further research would be necessary to select potential probiotic candidates from the fish external microbiome that can be used efficiently as biocontrol agents in a durable prophylactic management regime against columnaris disease.
